# Effect of Sinapine on Microstructure and Anti-Digestion Properties of Dual-Protein-Based Hydrogels

**DOI:** 10.3390/foods13203237

**Published:** 2024-10-11

**Authors:** Youdong Li, Mengxin Duan, Guoyan Liu, Li Liang, Xiaofang Liu, Jixian Zhang, Chaoting Wen, Xin Xu

**Affiliations:** 1 College of Food Science and Engineering, Yangzhou University, Yangzhou 225127, China; youdongli@yzu.edu.cn (Y.L.); 13053443229@163.com (M.D.); yzufsff@163.com (G.L.); liangli0508@hotmail.com (L.L.); zjx@yzu.edu.cn (J.Z.); chaoting@yzu.edu.cn (C.W.); 2 School of Tourism and Cuisine, Yangzhou University, Yangzhou 225127, China; liuxf@yzu.edu.cn

**Keywords:** cross-linked, sinapine, embedded delivery, in vitro digestion, delivery of hydrogels, drug delivery

## Abstract

Sinapine is a natural polyphenol from the cruciferous plant family that has anti-aging effects but is low in bioavailability. To improve the bioavailability and therapeutic effect of sinapine, sinapine-crosslinked dual-protein-based hydrogels were prepared using soy protein isolate as a cross-linking agent. The preparation conditions were optimized by single-factor experiments, and the optimal ratios were obtained as follows: the concentration of sinapine was 300 μg/mL; the water–oil ratio was 1:3. The encapsulation rate was greater than 95%, and the drug loading capacity was 3.5 mg/g. In vitro, digestion experiments showed that the dual-protein-based hydrogels as a drug carrier stabilized the release of sinapine and improved the bioavailability of sinapine by 19.3%. The IC50 of DPPH antioxidants was 25 μg/mL as determined by in vitro digestion, and the antioxidant capacity of ABTS was about 20% higher than that of glutaraldehyde control. This is due to the addition of sinapine to enhance the antioxidant properties of the system. It can be seen that the developed hydrogels have potential applications in related fields, such as food nutrition fortification and drug delivery.

## 1. Introduction

Sinapine is the main simple phenolic compound in rapeseed, with a content of approximately 1.2~2.3% [1]. It accounts for about 80% of the total phenol content of rapeseed, with a variety of biological activities, such as antioxidants, anti-radiation, anti-aging, blood pressure lowering, anti-inflammatory, anti-diarrhea, and anti-androgens [2,3]. However, orally-ingested sinapine has a low bioavailability due to its instability in gastrointestinal digestion and low permeability of small intestinal epithelial cells. Encapsulation of sinapine improves its tolerance to high temperature, neutral/alkaline environments, and promotes active transport of sinapine by small intestinal epithelial cells. Therefore, increasing its bioavailability and utilization through targeted delivery is conducive to expanding its application scope [4]. Feng et al. used protein and hyaluronic acid to prepare sinapine-supported nanoparticles to improve their bioavailability and effectively treat allergic asthma in guinea pigs [5]. Other researchers have made advances in the development of liposomes, microemulsions, and biomimetic cell membrane vesicles for the delivery of polyphenols [6,7,8]. However, they are generally faced with problems such as complex construction methods, high cost, and an unsuitability for oral administration. Therefore, a low-cost, simple, and efficient drug delivery strategy should be developed to enhance the potential of sinapine in disease.

Gelatin microspheres are a type of drug carrier prepared using animal protein, that has the advantages of simple preparation, low price, good dispersibility, good histocompatibility, and biodegradability. Gelatin has a negative charge and sinapine has a permanent positive charge, which can enhance their interaction through electrostatic adsorption [5,9,10,11,12]. Therefore, gelatin has the potential to be targeted as a carrier for the delivery of sinapine. The hydrogel system based on emulsion delivery systems can affect the digestion and absorption of plant active molecules in the following ways: firstly, improving the gastrointestinal solubility and stability of phytogenic actives, avoiding direct contact of the actives with the gastrointestinal environment, and reducing the inactivation of the actives; secondly, regulating the time-lagged release and specific adhesion behavior of the actives in the digestive tract.

Food-derived cross-linker construction system can make the system more stable. Using vegetable oil as the oil phase, the drug and gelatin in the water phase are combined to form a water-in-oil emulsion system by the emulsification cross-linking method, and a cross-linking agent is added to increase the viscosity of the system and to stabilize it, finally, the oil in the system is removed by isopropyl alcohol to obtain hydrogel [13,14]. However, commonly used cross-linking agents such as glutaraldehyde and EDA [12] face the disadvantages of strong toxicity and high price, thus, finding suitable alternatives can help prepare safer delivery products [15,16,17,18]. The function of the crosslinker is to transform the linear or mild branched-chain macromolecules into a three-dimensional network structure, to improve the strength, heat resistance, wear resistance [19], solvent resistance, and other properties [20]. Soy proteins as the main constituent of soy flour are long-chain polymer molecules with multiple reactive groups (−OH, −SH, −COOH, −NH_2_, etc.), which can be cross-linked with proper small or large molecular weight plasticizers. Soybean protein isolate (SPI) has lipophilic, hydrophilic, gelatinous, and emulsifying properties, which can enhance the stability of the solution. Therefore, it is necessary to select plant proteins as stabilizers to investigate the effects of the type of plant protein on the macroscopic properties of the hydrogel system and the loading rate of sinapine [21,22]. Studies on the bioactivity of sinapine have been widely reported, and the low bioavailability of sinapine is well known. However, few studies have attempted to build a system that improves its utilization. In our previous studies, we demonstrated the role of sinapine and the molecular self-assembly capabilities of proteins in the construction of oleo gels [23]. This provides the basis for constructing an anti-digestibility system for sinapine delivery.

In this research, the self-assembly of gelatin and sinapine molecules was developed to construct the condensate, and the interaction between SPI-gelatin and SPI-sinapine replaced the cross-linking agent to stabilize the system. The optimal conditions for preparing hydrogels were selected through single-factor experiments, and then the physicochemical properties and in vitro release properties were analyzed. The prepared hydrogel combines the carrier advantages of animal protein gelatin and the health advantages of vegetable protein, conforms to the concept of green and safe development, and creates favorable conditions for the production and industrial application of dual-protein composite gels. A sinapine administration strategy based on diprotein construction can improve the application potential of polyphenols in neuroinflammation, non-alcoholic fatty liver disease, and other diseases.

## 2. Materials and Methods

### 2.1. Materials

Sinapine was purchased from Chengdu DESITE Biological Co., Ltd. (Chengdu, China). SPI was purchased from Beijing SOLABAO Technology Co., Ltd. (Beijing, China). Gelatin was purchased from Shanghai Macklin Biochemical Co., Ltd. (Shanghai, China). 1,1-Diphenyl-2-picrylhydrazyl (DPPH) and 2,2′-Azinobis-(3-ethylbenzthiazoline-6-sulphonate) (ABTS) were purchased from Sigma (Shanghai, China). HCL, K_2_S_2_O_4_, K_2_HPO_4_, and NaCl (Analytically pure, ≥98%) were purchased from Shanghai Suke Chemical Co., Ltd. (Shanghai, China).

### 2.2. Methods

#### 2.2.1. Preparation of Gel

Gelatin was pre-soaked in room temperature water for 10 min and then prepared as 12 (*w*/*v*, %) gelatin solution under magnetic stirring and water bath heating at 50 °C. The aqueous phase was prepared by thoroughly mixing with an aqueous sinapine base solution in different proportions. The canola oil was processed into the oil phase by magnetic stirring in a 40 °C water bath. The aqueous phase was slowly dropped into the oil phase at a certain ratio of water to oil, stirred for 30 min, and then brought to 4 °C in an ice water bath. The water-in-oil emulsion was obtained. Then, isopropanol was added to the above mixture for vortex washing, centrifuged at 8000 r/min for 5 min, washed again, and then washed with water to obtain the de-oiled compound. Finally, 10 mL of 10 mg/mL SPI aqueous solution is added to the above complex and stirred thoroughly, stored in a refrigerator at 4 °C, and cross-linked and cured for 24 h, hydrogel was obtained after freeze-drying. After the single-factor optimization experiment was completed, the control group in the follow-up experiment was replaced by an equal concentration of glutaraldehyde, and other preparation conditions remained unchanged.

The specific protocols for the above single-factor experiments for the preparation of double-protein hydrogel are shown in Table 1.

#### 2.2.2. Determination of Drug Loading and Entrapment Efficiency of Double Protein Composite Gel

The standard curve of sinapine was established as follows: precisely weigh 1 mg of sinapine into a 100 mL volumetric flask, add 100 mL of deionized water, and dissolve to prepare a standard solution with a mass ratio of 1:1000 for future use. The UV absorption wavelength of sinapine is 326 nm. The standard aqueous solution of sinapine was diluted into six solutions at concentrations of 1:150, 1:300, 1:450, 1:600, 1:750, and 1:900 (*v*/*v*), respectively. The absorbance of each diluted solution at 326 nm (noted as A) was measured using a UV spectrophotometer. The equation of the standard curve for sinapine was obtained by linear regression of absorbance y on mass ratio x. Equation (1) is presented as follows:

Sinapine concentration (µg/mL) = 0.0133A − 0.0007(1)

Dissolve 0.1 g of blank double protein composite gel lyophilized sample in distilled water. Following ultrasonic treatment at 50 °C, the supernatant was centrifuged and the absorbance was measured at 326 nm. The absorbance of sinapine base loaded double protein composite gel was determined using the same method. The concentration of sinapine in the gelatin SPI gel loaded with sinapine was calculated based on the standard curve equation. The drug loading (2) and embedding rate (3) of sinapine loaded on the double protein gel were determined using the following formulas:

(2) $$\mathrm{Drug}\;\mathrm{loading}=\frac{\mathrm{actual}\;\mathrm{drug}\;\mathrm{amount}\;\mathrm{in}\;\mathrm{gels}}{\;\mathrm{total}\;\mathrm{mass}\;\mathrm{of}\;\mathrm{gels}}\times 100\%$$

(3) $$\mathrm{Embedding}\;\mathrm{rate}=\frac{\mathrm{actual}\;\mathrm{drug}\;\mathrm{amount}\;\mathrm{in}\;\mathrm{gels}}{\mathrm{drug}\;\mathrm{input}}\times 100\%$$

#### 2.2.3. Size Distribution and Zeta Potential Measurement

The particle size and potential of double protein gel emulsion were measured by a Mastersizer 1000 laser particle size analyzer (model Zetasizer Es 90 Nano, Malvern Panalytical, Malvern, UK). The measurement steps are as follows: put the sample into the Hydro 2000SM sampling device until the shading rate reaches 8%, and obtain the particle size distribution and surface weighted average diameter. Dilute the sample 100 times and measure the particle surface charge (Zeta potential) with a Nano ZS nano laser particle sizer.

#### 2.2.4. Morphological Analysis of Double Protein Composite Gel

The emulsion formation state was observed by an optical microscope as follows: take a small amount of emulsion to the glass slide and cover the cover glass slide, respectively at 100× and 400×, and observe the emulsion after magnetic stirring and vortex treatment.

#### 2.2.5. Determination of Morphology of Double Protein Composite Gel

To understand the effect of sinapine as a cross-linking agent on the formation of diprotein hydrogels, the apparent morphological characteristics were observed by FESEM as follows. A small amount of lyophilized hydrogel powder was placed on the conductive adhesive to make a corresponding FESEM sample. After sputtering, the surface morphology of the hydrogel was observed using FESEM (Nova Navo SEM 450, FEI Co., Hillsboro, OR, USA).

#### 2.2.6. Determination of the Internal Structure of Double Protein Composite Gel

To understand the interaction and localization of protein and sinapine in the hydrogel system, TEM was performed as follows. The lyophilized hydrogels were redistributed in water (1 mg/mL) for 10 min by ultrasonic treatment, and a drop of dispersion was dropped on the copper network and dried under infrared light. TEM (jem-2100, JEOL Co., Akishima, Japan) was used to observe the internal structure of the compound protein gel.

#### 2.2.7. Swelling Degree of Gel

For measuring the swelling degree of a gel, dissolve an appropriate amount of compound protein gel in a phosphate buffer solution with a pH of 7.4 as solvent. Place it at room temperature for 24 h, centrifuge the solution, remove the sediment and weigh it after the water on the surface of the gel is sucked dry. Repeat the experiment three times. Calculating the swelling degree of compound protein gel can be performed according to the following Formula (4):

(4) $$\mathrm{Swelling}\;\mathrm{degree}=\frac{\mathrm{mass}\;\mathrm{of}\;\mathrm{gel}\;\mathrm{after}\;\mathrm{swelling}-\mathrm{mass}\;\mathrm{of}\;\mathrm{dried}\;\mathrm{gel}}{\mathrm{mass}\;\mathrm{of}\;\mathrm{dried}\;\mathrm{gel}}\times \;100$$

#### 2.2.8. FT-IR Analysis

The intermolecular force was observed by using FT-IR. An FT-IR (Nicolet 200SXV) spectrometer was used to analyze the structural composition and interaction of the sinapine-loaded double protein composite gel samples. The obtained IR spectra were used to determine the possible interactions between cross-linked sinapine, gelatin molecules, and functional groups in SPI [24].

#### 2.2.9. Determination of Sinapine Release Rate In Vitro

The gastric juice was prepared by mixing 3.2 mg/mL pepsin and 2 mg/mL sodium chloride. The intestinal fluid was prepared by mixing 6.8 mg/mL K_2_HPO_4_, 0.8 mg/mL intestinal protease, 20 mg/mL bile salt, and 8.8 mg/mL sodium chloride. Lyophilized hydrogels were dissolved in deionized water and added to gastric juice for a full reaction. Samples were taken in gastric juice and intestinal juice every 10 min.

The UV-5000 ultraviolet-visible spectrophotometer was used to measure the absorbance value at 326 nm, and the concentration of sinapine was calculated according to the standard curve. To calculate the cumulative release rate of bioactive factors the following formula is applied (5):

(5) $$\mathrm{Cumulative}\;\mathrm{release}\;\mathrm{rate}\;\mathrm{of}\;\mathrm{the}\;\mathrm{hydrogel}=\frac{\sum_{i=1}^{n}W_{i}}{\mathrm{Total}\;\mathrm{drug}\;\mathrm{loading}\;\mathrm{in}\;\mathrm{hydrogel}}\times 100\%\;$$

where W_i_ is the cumulative drug release at each sampling time.

#### 2.2.10. In Vitro Antioxidant Analysis of Double Protein Composite Gel

The ABTS cationic radical working solution was obtained by diluting with pure water until the absorbance of the basic solution measured at 734 nm was 0.7 ± 0.005. To follow, accurately pipette 0.1 mL of the sample solution, add 3.9 mL of the working solution, and mix well. Immediately after incubation at 25 °C for 6 min, the absorbance value (A) was measured at 734 nm and the methanol solution was used as a blank control (A_0_). the radical scavenging ability of ABTS was calculated according to Equation (6). DPPH antioxidant assay was developed with the following steps: 2 mL of sample solution and 2 mL of 300 μmol/L prepared DPPH solution was added. After 30 min of reaction in the dark, absorbance was measured at 517 nm. Each group of samples was measured three times in parallel, and the DPPH radical scavenging ability of the samples was calculated according to the following Equation (7):

(6) $$\mathrm{ABTS}\;\mathrm{radical}\;\mathrm{scavenging}\;\mathrm{ability}\;(\%)=\left(1-\frac{A}{A_{0}}\right)\times 100\%$$

(7) $$\mathrm{DPPH}\;\mathrm{radical}\;\mathrm{scavenging}\;\mathrm{ability}\;(\%)=\left(1-\frac{A_{\mathrm{sample}}-A_{\mathrm{Blank}}}{A_{\mathrm{contrast}}}\right)\times 100\%$$

#### 2.2.11. Statistical Analyses

Origin 9.0 (Origin Lab Corporation, Northampton, MA, USA) statistical software was used for statistical analysis. All experimental data were expressed as the mean ± standard deviation (SD). Differences between the test subjects and model controls were evaluated using Student’s *t*-test (the test sample n = 5 was repeated 3 times, *p* < 0.05).

## 3. Result and Discussion

### 3.1. Effect of Sinapine Concentration on the Particle Size of Gel

**Figure 1:**
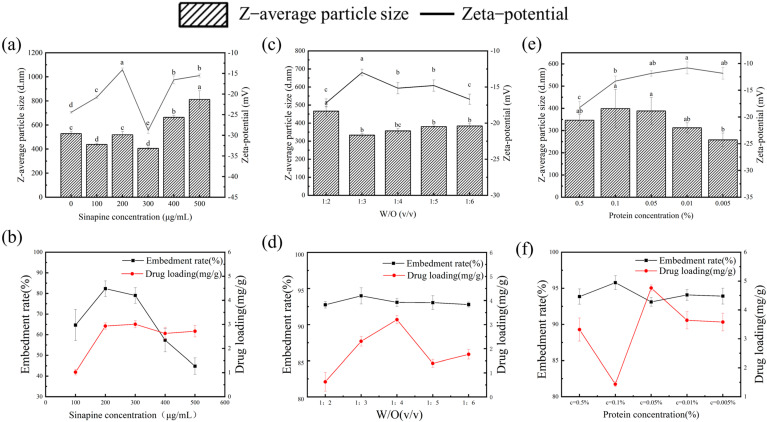
Effects of different water–oil ratios, sinapine concentrations, and protein concentrations on particle size potential, drug loading, and entrapment rate of gel (**a**,**b**) W/O; (**c**,**d**) sinapine concentration; (**e**,**f**) protein concentration. Different letters above each column represent significant differences (*p* < 0.05).

Hydrogels are one of the most popular and represented biomaterials in tissue engineering due to their biocompatibility and functionality, as well as their similarity to biological soft tissues. Hydrogels have been widely used in bioengineering applications, such as controlled drug delivery systems. First, the encapsulation rate and drug loading capacity of bioactive substances are critical to the functional properties of hydrogels. Secondly, the particle size potential of hydrogels is also an important indicator of stability. In this experiment, the effect of different concentrations (500 μg/mL, 400 μg/mL, 300 μg/mL, 200 μg/mL, and 100 μg/mL) of sinapine on the particle size potential, drug loading, and encapsulation rate was investigated using univariate experiments. As shown in Figure 1a, with increasing sinapine concentration, greater adsorption of gelatin results in an increased droplet size of the emulsion and a larger final hydrogel size. However, as shown in Figure 1b, as the concentration of sinapine increased, the encapsulation rate initially rises then decreases, with the maximum encapsulation rate at 300 μg/mL. This may be attributed to the optimal binding of sinapine to gelatin at this concentration, and as the sinapine concentration increases, the gelatin may not be sufficient to bind excess sinapine, resulting in a washout of the system, which in turn decreases the embedding rate of the hydrogel. The minimum particle size remained stable and the embedding rate was highest at 300 μg/mL; thus, the concentration of 300 μg/mL was selected.

### 3.2. Effect of Water-Oil Ratio on Particle Size of Gel

The effects of different water–oil ratios of 1:2; 1:3; 1:4; 1:5; and 1:6 on the particle size potential, drug load, and encapsulation rate were investigated experimentally. As presented in Figure 1d, with a decrease of the water–oil ratio, the entrapment rate of sinapine decreases, which may be attributable to the decrease of dispersion of emulsion with the increase in oil phase volume, resulting in a decreased entrapment rate of the gels. As shown in Figure 1c, the particle size of the gels is the smallest at 1:3, which shows an increasing trend, indicating that as the oil phase volume increases, the adhesion of the gels increases, resulting in a larger particle size [25]. Moreover, the more vegetable oil required in the preparation of the hydrogel, the more detergent is needed for cleaning the gels, which also increases the preparation cost. Considering all these aspects, the water–oil of 1:3 was selected as the best value.

### 3.3. Effect of Protein Concentration on Gel

The effects of different protein concentrations of 0.5%, 0.1%, 0.05%, 0.01%, and 0.005%, on the particle size, drug load, and embedding rate of different proteins were investigated experimentally. As shown in Figure 1e, as the protein concentration decreases, the particle size of the gels increases first and then decreases, the particle size is the largest at 0.1% which may be due to the reduced protein concentration, the degree of cross-linking between the beads is also reduced, which is more conducive to a spherical shape, resulting in smaller particle size. As shown in Figure 1f, the embedding rate is the largest at 0.1%, indicating a better embedding effect of sinapine, which results in the largest particle size [26].

### 3.4. Determination of Particle Size

Glutaraldehyde can accumulate in the human body after cross-linking with proteins and, with age, becomes lipofuscin, which is a sign of aging. In this study, SPI was used as a cross-linking agent to prepare hydrogels with a smaller particle size of about 500 nm, when the particle size of the glutaraldehyde control group was 1.7 μm. The results indicate that SPI could significantly reduce the particle size of the gel. From Table 2, it can be found that the zeta potential of the glutaraldehyde control group was stronger, with a potential of −4.86 ± 0.15 mv. The positive charge of the gelatin was shielded due to the cross-linking of the aldehyde group (-CHO) of glutaraldehyde with the positively charged amine group (-NH_2_) in the gelatin [27].

### 3.5. The Internal Structure of the Gel

**Figure 2:**
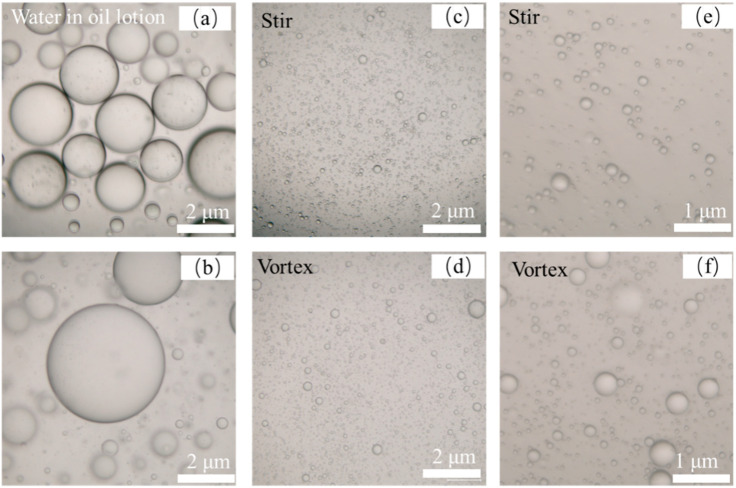
Optical micrograph of emulsion without the protein crosslinker (**a**,**b**) and different emulsification methods: (**c**,**e**) stir; (**d**,**f**) vortex.

Through the observation of the morphology of emulsion by optical microscope, as shown in Figure 2a,b, it can be observed that the particle size of emulsion without a protein cross-linking agent is larger and the particle size of emulsion is reduced to a certain extent by stirring vortex as shown in Figure 2d,f after cross-linking, and the particle size after magnetic stirring as shown in Figure 2c,e is more uniform.

**Figure 3:**
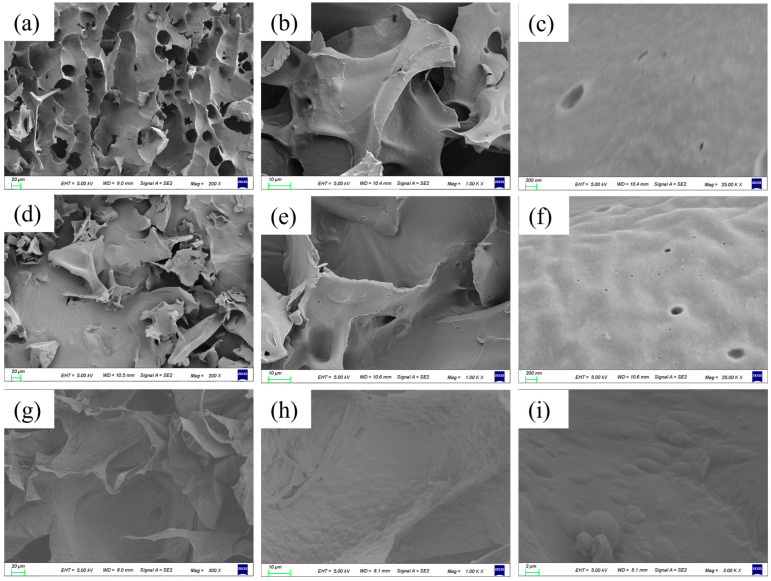
SEM photo of sinapine-loaded double protein composite gel. (**a**–**c**) No sinapine was added and the protein concentration was 1.5%; (**d**–**f**) sinapine was added and the protein concentration was 1.5%; (**g**–**i**) added sinapine and protein concentration was 0.5%.

Scanning electron microscopy presented in Figure 3a–c shows the hydrogel prepared with a 1.5% protein crosslinker concentration without embedding sinapine. It can be seen that there are large holes on the surface of the hydrogel, and the surface is smoother after further amplified. Figure 3d–f shows the hydrogel with a 1.5% protein concentration and embedded meson base. It can be observed that the hydrogel presented fragmentation, and the addition of sinapine filled the pores, resulting in irregular edges on the fragments. After further amplification, the hydrogel surface had a slight bulge, indicating that sinapine is embedded in the influent gel, filling the gap to some extent. Figure 3g–i shows the hydrogel loaded with sinapine prepared with a 0.5% protein concentration as a cross-linking agent. When the protein concentration decreases, the surface of the hydrogel becomes softer, the edges are neat, and the surface has spherical bulges. The morphology of sinapine gradually appears. By comparing whether the first two groups are loaded with sinapine, it can be concluded that the addition of sinapine will make the hydrogel system fuller and it also proves that the dual protein can prepare porous hydrogels for embedding active substances. Compared with the latter two groups, the concentration of the protein cross-linking agent has a certain regulatory effect on the digestion and release time of sinapine in vivo [28].

**Figure 4:**
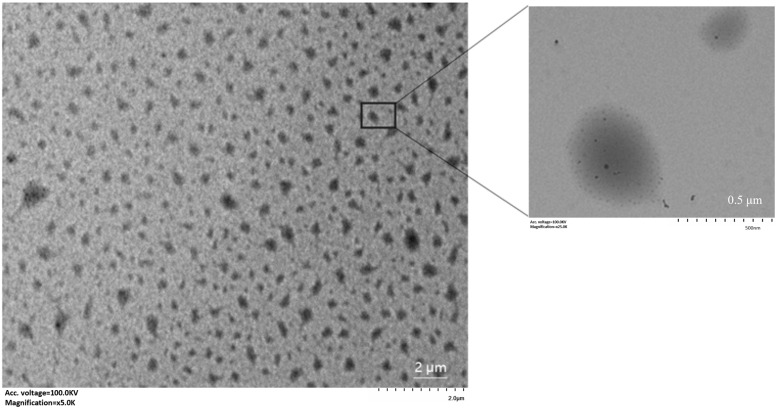
TEM photo of sinapine-loaded double protein composite gel.

The hydrogels were diluted with water and observed by transmission electron microscopy. As shown in Figure 4, the emulsion was irregularly rounded and spherical with good dispersion. After magnification, it can be seen that SPI is attached to the surface of the gelatin ball and loaded with tiny sinapine particles. This is consistent with the results from scanning electron microscopy.

### 3.6. Effect of Swelling Degree of Gel

**Figure 5:**
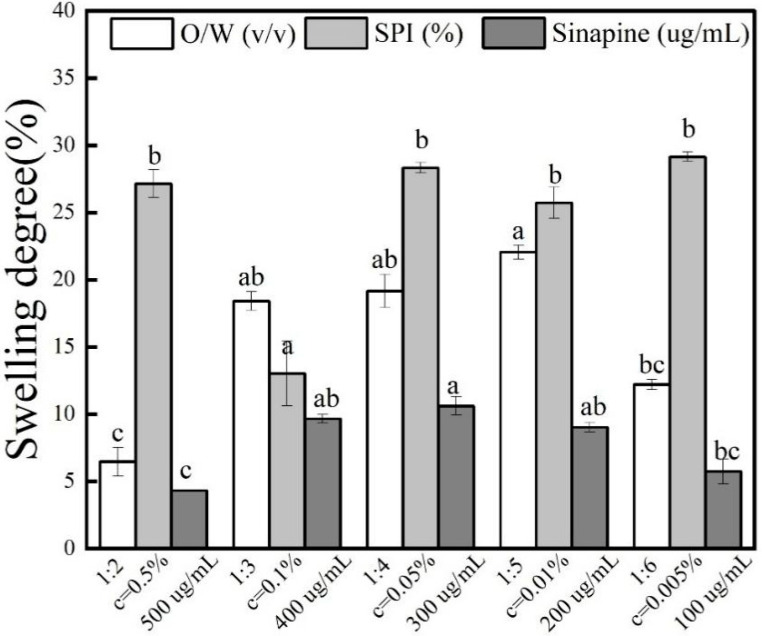
Swelling degree of sinapine loaded double protein composite gel (water–oil ratio: 1:2, 1:3, 1:4, 1:5, 1:6; protein concentration: 0.5%, 0.1%, 0.05%, 0.01%, 0.005%; the concentration of sinapine was 100, 200, 300, 400, 500 μg/mL). Different letters above each column represent significant differences (*p* < 0.05).

After placing the gel in a phosphate buffer solution for 24 h, the swelling degree was calculated. It can be seen from Figure 5 that with the increase of the oil phase volume and sinapine concentration, the swelling degree increases first and then decreases. Some studies have shown that cross-linking may reduce the swelling rate of the gel. Bigi et al. found that with an increase in the degree of intermolecular cross-linking, the swelling rate showed a significant negative trend [29]. Researchers used GTA (Gelatin Tea Extract) to modify the gelatin molecules. The results indicated that the addition of polyphenols increased the degree of cross-linking and reduced the degree of swelling. As can be seen from the detailed data presented in Table 3, the swelling of the hydrogel is minimal at about 8% under optimal preparation conditions. This is because gelatin, sinapine, and SPI slowly shrink during the curing process to squeeze out the water in the gel. It still has numerous microchannels in the gel. With the increase of sinapine concentration, the strength of cross-linking with gelatin was greater, and the gel formed was more stable. The greater the density of the gelatin skeleton in the cross-linking process, the stronger the resulting structure. Therefore, as the pore diameter of the composite gel within the material channel decreases, the concentration of OH groups and amidogen per unit volume increases, and the stronger the water absorption capacity, the greater the expansibility. Simultaneously, due to the reduction in the microchannel aperture, the amount of water entering the microchannel diminishes. Consequently, the drug release speed is slower, resulting in a better release effect.

### 3.7. Cross Linking Mechanism between Sinapine, Gelatin, and Soy Protein Isolate

**Figure 6:**
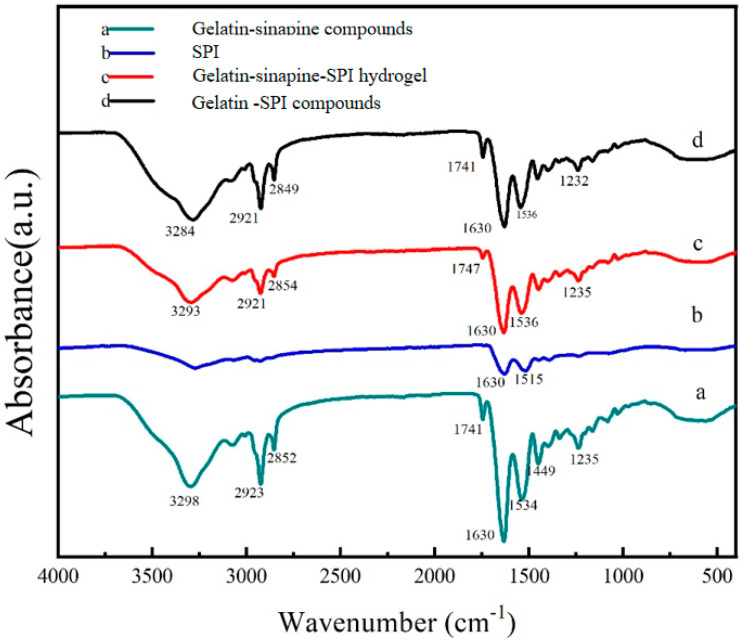
FT-IR of sinapine-loaded double protein composite gel.

When the gelatin solution is at a temperature lower than 35–40 °C, the collagen molecule spirochete denatures and decomposes into a single polypeptide chain, changing from a triple helix to a single helix, forming a reversible gel micelle. Therefore, under the influence of temperature fluctuation, gelatin can easily recover to the triple helix structure of collagen. In addition, when gelatin is in a gel state, its physical network structure will decompose at high temperatures, which is unsuitable for long-term use at 35–40 °C. Due to the low thermal and mechanical stability of gelatin hydrogels, suitable methods should be used for cross-linking [30]. By analyzing the Fourier infrared spectrum as shown in Figure 6, from the bottom to top, the hydrogel without SPI, double protein composite gel loaded with sinapine, and blank double protein composite gel are ordered. The FT-IR spectrum shows the conformational relationship between the tyrosine of the gelatin molecule and the main chain of SPI; this can be used to explain the structure of gelatin in the presence of the cross-linking agent and the interaction between the three raw materials. One noticeable difference is that the sample gels shifted from 3293 cm^−1^ to a lower band and a wider band in the IR spectrum c and d of -OH peaks compared to the gels without sinapine (Figure 6) [31]. It has been recognized that interactions through hydrogen bonding between the chemical groups of compounds can cause a peak shift to lower wavenumbers [28]; therefore, the shift of amide I and II bands and the wider peak after incorporation of TA indicated the presence of hydrogen bonding [21]. The absorption peak at wavelength 2810–2880 cm^−1^ corresponds to (-CH_2_) asymmetric stretching. According to the literature survey, the interaction between amino and carboxyl groups of soybean and gel may indicate that the mechanical properties of the composite fiber are improved, thus indicating that hydrogen bonding is the main mechanism causing these interactions [32].

### 3.8. In Vitro Release and Antioxidant Analysis of Gel

**Figure 7:**
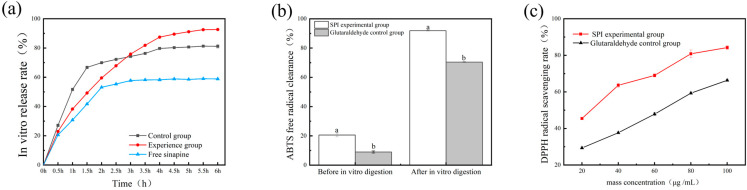
In vitro digestibility and antioxidant properties analysis of dual-protein-based hydrogels. (**a**) In vitro digestibility of free sinapine and sinapine-crosslinked dual-protein-based hydrogels; (**b**) antioxidant capacity of ABTS after in vitro digestion; (**c**) antioxidant capacity of DPPH after in vitro digestion. Different letters above each column represent significant differences (*p* < 0.05).

After 6 h of gastrointestinal digestion in vitro as shown in Figure 7a, the bioavailability of sinapine alone in vivo is low. After 2 h of gastric digestion, sinapine was digested and decomposed into erucic acid; the entrapped sinapine was slowly released in intestinal juice after gastric digestion, and the release rate reached about 90%. In the control group using glutaraldehyde as a cross-linking agent, sinapine can also be released slowly in the small intestine, and the release rate is about 80%. Thus, the dual-protein composite gel served as a carrier to improve the bioavailability of sinapine.

Sinapine as a plant polyphenol was evaluated in this study to explore the practical implications of the antioxidant capacity of the loaded sinapine bioprotein before and after in vitro digestion. After in vitro gastrointestinal digestion, the dual-protein complex gel delivery system was dispersed. The system was broken down in the stomach by gastric acid and then immersed in intestinal fluid, where the soybean isolate protein and gelatin cross-linked decreased, and sinapine was slowly released from the stable system.

Figure 7b shows the free radical scavenging ability of ABTS before and after in vitro digestion of the sinapine-crosslinked dual-protein-based hydrogels group and the glutaraldehyde control group. It is evident that before in vitro digestion, the ABTS antioxidant capacity of the experimental group of soybean isolate protein was 10% higher than that of the glutaraldehyde control group; after in vitro digestion, the experimental group of soybean isolate protein was 20% higher than that of the glutaraldehyde control group, indicating that the soybean isolate protein showed better encapsulation effect. In addition, the scavenging ability of DPPH radicals was also measured. As presented in Figure 7c, the soy isolate protein group showed better release after in vitro digestion than the glutaraldehyde control group. The IC50 of DPPH antioxidants was 25 μg/mL. It can be observed that the double-protein gel loaded with sinapine has a strong antioxidant capacity after in vitro digestion. The scavenging ability of sinapine was stronger compared to the control group.

## 4. Conclusions

The following conclusions can be drawn from studying the properties and structure of the sinapine-crosslinked dual-protein-based hydrogels prepared with SPI as the cross-linking agent. The encapsulation rate of the optimized hydrogel sinapine was greater than 95% and the drug loading was 3.5 mg/g. Moreover, the addition of sinapine improved the antioxidant activity and bioavailability of sinapine. FT-IR spectroscopy confirmed that the interaction between gelatin chains was closely enhanced by the cross-linking of hydrogen bonds between the two proteins, while the conformation of gelatin was not changed. This study has only verified its performance in vitro; thus, future in vivo experiments are required. Our research has developed a water-soluble active ingredient delivery hydrogel, and the results provide a basis for developing a new delivery system for food processing and biomaterials.

## Figures and Tables

**Table 1:** Specific experimental protocol for single-factor experiments with double-protein hydrogel.

Test	Factors		
	W/O (*v*/*v*)	SPI (%)	Sinapine Concentration (μg/mL)
1	1:2	0.5	100
2	1:3	0.1	200
3	1:4	0.05	300
4	1:5	0.01	400
5	1:6	0.005	500

**Table 2:** Comparison of particle size potential between the glutaraldehyde control group and SPI experimental group. ^a^ and ^b^ indicate significant differences between groups (*p* < 0.05).

Sample	Size (nm)	Zeta Potential (mV)
Glutaraldehyde control group	1780.03 ± 31.43 ^a^	4.86 ± 0.15 ^a^
SPI experimental group	500.53 ± 11.24 ^b^	3.69 ± 0.13 ^b^

**Table 3:** Swelling degree of gel loaded with sinapine. Different letters indicate significant differences between groups (*p* < 0.05).

Sample	Sinapine Concentration	W/O	SPI Concentration	Swelling Degree
	(μg/mL)	(*v*/*v*)	(%)	
1	500	1:3	0.01	4.35 ± 1.03 ^c^
2	400	1:3	0.01	10.18 ± 0.32 ^ab^
3	300	1:3	0.01	8.17 ± 0.68 ^a^
4	200	1:3	0.01	6.32 ± 0.35 ^ab^
5	100	1:3	0.01	4.69 ± 0.91 ^bc^
6	300	1:2	0.5	5.6 ± 1.03 ^c^
7	300	1:3	0.5	17.73 ± 0.4 ^ab^
8	300	1:4	0.5	17.54 ± 0.4 ^ab^
9	300	1:5	0.5	16.52 ± 1.17 ^a^
10	300	1:6	0.5	17.95 ± 0.35 ^bc^
11	300	1:3	0.5	17.88 ± 1.06 ^b^
12	300	1:3	0.1	14.58 ± 0.7 ^a^
13	300	1:3	0.05	26.53 ± 1.24 ^b^
14	300	1:3	0.01	8.17 ± 0.68 ^b^
15	300	1:3	0.005	29.46 ± 0.37 ^b^

## Data Availability

The original contributions presented in the study are included in the article, further inquiries can be directed to the corresponding author.
